# N-acetyl cysteine for the treatment of alcohol use disorder: study protocol for a multi-site, double-blind randomised controlled trial (NAC-AUD study)

**DOI:** 10.1136/bmjopen-2024-091631

**Published:** 2025-09-08

**Authors:** Kirsten Morley, Shalini Arunogiri, Jason P Connor, Paul J Clark, Mary Lou Chatterton, Andrew Baillie, Tim Slade, Michael Berk, D Lubman, Paul S Haber

**Affiliations:** 1 Specialty of Addiction Medicine, The University of Sydney School of Medicine, Sydney, New South Wales, Australia; 2 Eastern Health Clinical School, Monash University, Box Hill, Victoria, Australia; 3 The University of Queensland, Saint Lucia, Queensland, Australia; 4 Department of Gastroenterology and Hepatology, Princess Alexandra Hospital, Brisbane, Queensland, Australia; 5 Monash University, Clayton, New South Wales, Australia; 6 Health Sciences, University of Sydney, Camperdown, New South Wales, Australia; 7 Matilda Centre, The University of Sydney, Sydney, New South Wales, Australia; 8 Psychiatry, Deakin University Faculty of Health Medicine Nursing and Behavioural Sciences, Geelong, Victoria, Australia; 9 Drug Health Services, Sydney Local Health District, Camperdown, New South Wales, Australia

**Keywords:** MENTAL HEALTH, PSYCHIATRY, Substance misuse

## Abstract

**Introduction:**

Current treatments for alcohol use disorders (AUD) have limited efficacy. A previous 28-day pilot trial of N-acetyl cysteine (NAC) vs placebo found NAC to be feasible and safe, with evidence of improvement on some measures of alcohol consumption. Thus, the primary aim of the NAC-AUD study is to examine the therapeutic and cost-effectiveness of NAC vs placebo in improving treatment outcomes for AUD. We will also examine the (i) effect of NAC vs placebo on mood, markers of liver injury, cognition and hangover symptoms; and (ii) predictors of any response.

**Methods and analysis:**

This double-blind trial will randomise participants with AUD to a 12-week regimen of either NAC (2400 mg/day) or placebo. All participants will receive medical management. The primary drinking outcome will be the number of heavy drinking days (HDDs) per week, validated by phosphatidylethanol (PEth). Secondary alcohol-related outcomes will include standard drinks per drinking day (SDDD) per week and absence of any HDDs. Other secondary outcomes will include markers of liver injury, depression, anxiety, craving, hangover symptoms, cognition and blood oxidative stress markers. We will also examine the cost-efficacy of NAC vs placebo.

**Ethics and dissemination:**

Ethics approval for the study has been granted by The Sydney Local Health District Ethics Review Committee (X21-0342& HREC2021/ETH11614). There are no restrictions on publication from the sponsor or other parties.

**Trial registration number:**

NCT05408247.

**STRENGTHS AND LIMITATIONS OF THIS STUDY**

- First well-powered multi-site study using a double-blind randomised controlled design to assess the therapeutic and cost-effectiveness of N-acetyl cysteine (NAC) for the treatment of alcohol use disorder (AUD).
- The trial will also examine the effectiveness of NAC on a range of salient comorbid conditions and symptoms associated with AUD, including liver disease, mood, sleep, hangover and cognition.
- This trial will use the gold standard objective biomarker phosphatidylethanol to validate alcohol consumption data.
- Examined the effectiveness of a potential medication that may be suitable for comorbidities in AUD.
- Stratification of relevant characteristics and measurement of secondary outcomes may provide clinically important information about patient groups that are likely to benefit from NAC treatment. However, subgroup analyses may not be sufficiently powered to determine a personalised approach to treatment.

## Introduction

 Alcohol misuse is a significant concern worldwide, being implicated in many physical and mental health conditions and accounting for approximately 5.3% of total global mortality.^1^ Alcohol use disorder (AUD) is defined as a pattern of heavy and excessive alcohol consumption that leads to negative biological, behavioural, health and social consequences.^2^ There are currently several pharmacotherapies available to treat the core symptoms of AUD and reduce consumption.^3^ However, the effectiveness of these pharmacotherapies is generally modest^4^ and they are not suitable for patients with alcohol-related liver disease (ArLD) which is a common comorbidity with high mortality.^5^ Emerging medications that are generally safe for those with liver disease, such as baclofen, may not be appropriate in patients with common comorbid psychiatric conditions and those at risk of overdose.^6 7^ Other emerging medications, such as topiramate, which might be at least as effective if not superior to front-line naltrexone,^8^ may not be appropriate for patients with physical complications such as liver disease, kidney disease or other risks for metabolic acidosis. Further development of new and effective treatments suitable for a diverse range of patients with AUD and their complexities is urgently required.

N-acetyl cysteine (NAC) is an over-the-counter antioxidant, is widely used by intravenous infusion for the treatment of acetaminophen overdose and may be a potential treatment for AUD.^9^ NAC has been demonstrated to replenish the intracellular levels of glutathione (GSH), a key antioxidant synthesised in cells and interacts with reactive oxygen species.^10^ Oxidative stress and neuroinflammation are tightly associated with chronic alcohol intake and relapse.^11^ AUD individuals demonstrate elevated oxidative stress markers and an inflammatory cytokine profile^12^,^14^ and which have also been observed to be predictive of future heavy drinking.^15^ NAC is also thought to normalise expression of the glutamate transporter (GLT-1) and cystine-glutamate exchange, restoring glutamate homeostasis, a neurotransmitter thought critical in the development and persistence of severe AUD. Chronic alcohol intake leads to reduced GLT-1 protein levels and disruption of glutamatergic input from the prefrontal cortex to reward areas.^16^ To this end, NAC has been demonstrated to modulate alcohol-related behaviours across a range of animal models, including (i) ethanol self-administration and intake,^17 18^ (ii) symptoms of ethanol withdrawal^19^ and (iii) alcohol reacquisition following abstinence from ethanol.^17 18^ Furthermore, preclinical studies neatly show that NAC administration inhibits neuroinflammation induced by chronic ethanol intake^19^ and fully abolishes elevated ethanol-induced oxidative stress.^11^

Clinical studies involving a range of neuropsychiatric disorders have demonstrated that NAC is well tolerated and has a good safety profile.^20^ Signs of efficacy have been observed for disorders similarly associated with impulse control including gambling disorder^21^ and skin picking,^22^ although these studies were relatively small. There have been several randomised controlled trials in substance use disorders with mixed results. For example, trials have reported decreases in daily smoking and alleviation of depression in tobacco use disorder,^23^ improvements of abstinence and dependence severity in cocaine users,^24^ but no improvements in any clinically relevant measure for methamphetamine dependence.^25^ One trial of NAC to optimise methadone maintenance therapy for individuals with substance use disorder reported significant improvements in anxiety and depression.^26^ Meta-analyses have concluded that NAC may provide some benefits in attenuating substance craving and might help alleviate symptoms associated with depression and alcohol withdrawal syndrome.^27^

There have been no full-length randomised controlled trials (RCTs) of NAC in patients seeking treatment for AUD to date, although studies among individuals without AUD indicate some promise. In a human laboratory study, Coppersmith *et al*^28^ examined relatively low dose (600–1800 mg) of NAC vs placebo on mitigating hangover symptoms in healthy controls and reported improvement after NAC vs placebo among female participants. A secondary analysis of alcohol consumption in individuals with cannabis use disorder randomised to NAC (2400 mg/day) vs placebo found significant increases in alcohol abstinence and reduction in weekly drinking days for NAC vs placebo.^29^

Our team recently conducted a proof-of-concept pilot trial of NAC vs placebo over 28 days among heavy drinking, treatment-seeking individuals with AUD, specifically reporting on feasibility and clinical outcomes in an outpatient context.^30^ We reported that NAC appears to be feasible and safe, with no significant differences in overall retention between treatment groups and minimal adverse events. There was a significant effect of NAC vs placebo on some measures of alcohol consumption; however, a variable pattern of effect across time indicated that a larger trial for a longer treatment duration was required to determine efficacy.

Accordingly, the NAC-AUD study aims to be the first, well-powered double-blind, randomised trial that examines the treatment and cost-effectiveness of NAC vs placebo (PL) in improving outcomes in active drinkers. The trial also aims to examine the effectiveness of NAC vs PL on a range of salient comorbid conditions and symptoms associated with AUD, including liver disease, mood, sleep, hangover and cognition. Specifically, we hypothesise that (i) NAC-treated participants compared with PL will significantly reduce their alcohol consumption, with the primary outcome measure being the number of heavy drinking days and secondary alcohol measures being drinks per drinking day and absence of heavy drinking day (HDD); (ii) NAC treatment will be more cost-effective than PL; (iii) comorbid clinical conditions (eg, alcohol-related liver disease, depression, anxiety, sleep disturbances and suicidal ideation), hangover symptoms, cognitive functioning and markers of oxidative stress (such as glutathione peroxidase (GPx) activity in blood cells) will be significantly improved in NAC-treated patients compared with PL; and (iii) beneficial NAC treatment response will be associated with blood markers of oxidative stress and comorbid clinical condition severity (eg, alcohol-related liver disease, depression).

The current article presents the protocol for the NAC-AUD RCT whereby prospective reporting contributes to clinical trial quality^31^ and is written to comply with the recommended Standard Protocol Items: Recommendations for Interventional Trials guidelines for RCT protocols.^32^ Results of this study will add to the current alcohol pharmacotherapy literature by providing high-quality data regarding the efficacy and cost-efficacy of this low cost and accessible medication. While the use of an objective laboratory marker of alcohol use is not novel and has been recommended for all alcohol treatment trials,^33^ this will be the first NAC trial to use phosphatidylethanol for this purpose. Furthermore, the secondary outcomes to be evaluated in this trial will provide clinically important information about patient groups that are likely to benefit from NAC treatment. Improving the therapeutic benefit of pharmacotherapy for AUD remains a challenge particularly given the presence of comorbidities that may be contra-indicated for certain medications. The NAC-AUD trial thus has the potential to facilitate the development of alternate pharmacotherapy options for AUD, including those with multiple comorbidities.

## Methods and analysis

### Study objectives

We are conducting a 12-week, double-blind, randomised controlled trial to examine the effectiveness of N-acetyl-cysteine (NAC, 2400 mg/day) vs PL in improving treatment outcomes in heavy drinkers.

### Patient recruitment

The trial is being conducted in several outpatient hospital and community settings across New South Wales, Queensland and Victoria, Australia. We aim to recruit up to 180 participants on a rolling basis through clinical referral (from treating clinicians of participating hospitals and community clinics) and via flyers/community advertisements in general practices, newspapers, websites (eg, Gumtree) and social media (eg, Facebook). Based on our previous experience, recruitment from mixed sources does not significantly impact treatment retention or outcome in alcohol pharmacotherapy trials.^34^ Participant time and travel expenses are reimbursed at the follow-up research assessments.

### Randomisation and allocation concealment

The study is a double-blind, RCT with participants randomised to receive one of two possible medication arms. The study is conducted under double-blind conditions so that participants and study staff are unaware of medication assignment. A computer-generated random allocation to NAC or placebo will be provided by an independent service inbuilt to RedCap software and stratified by site, smoking, sex, alcohol-related liver disease and medications for mood disorders. This is provided to the Clinical Trials Pharmacist at each site for intervention allocation and medication dispensing. In the event of a medical emergency that requires knowledge of the treatment condition, the investigators can contact the pharmacist to break the randomisation code for that individual.

### Inclusion and exclusion criteria

*Inclusion criteria:* (a) age 18 to 70 years; (b) alcohol use disorder according to Diagnostic and Statistical Manual of Mental Disorders, Fifth Edition (DSM-5) criteria; (c) a desire to reduce or stop drinking; (d) adequate cognition and English language skills to give valid consent and complete research interviews; (e) average weekly alcohol consumption of >21 standard drinks, with a weekly average of >2 heavy drinking days (HDDs: ≥5 standard drinks/day for men; ≥4 for women) during the month before screening; (f) written informed consent; (g) stable housing.

*Exclusion criteria:* (a) pregnancy or lactation—women are advised to use reliable contraception for the duration of drug therapy and a urine pregnancy test is performed as appropriate; (b) concurrent use of any psychotropic medication other than antidepressants (provided that these are taken at stable doses for at least 2 months); (c) any substance use disorder other than nicotine; (d) clinically unstable systemic medical condition (eg, cancer, end-stage liver disease: for example, Model for End-stage Liver Disease score ≥10 or clinician judgement) or psychiatric disorder (eg, active psychosis, borderline personality disorder, active suicide risk—as assessed by Structured Clinical Interview for DSM-5 – Research Version [SCID-5-RV] or clinician judgement) that precludes trial participation; (e) concurrent use of selenium, vitamin D or other antioxidants; (f) any alcohol pharmacotherapy within the past month.

### Pharmacotherapy schedule

The dosage of NAC is 2400 mg/day in two divided daily doses of 1200 mg (2×600 mg capsules) determined by the following: (i) greater signals of efficacy occur with 2400 mg vs lower doses without any difference in the adverse event profile; and (ii) two divided daily doses of 1200 mg (2×600 mg capsules) were administered in the pilot trial by this team yielding early signals of efficacy.^30^ NAC is available over the counter in Australia and was purchased from PharmaZell in Germany. The optimal comparator group is placebo given that the NAC-AUD trial will include comorbidities such as alcohol-related liver disease that may be unsuitable for currently available ‘active’ comparator pharmacotherapies. The placebo used in this trial is dicalcium phosphate, given the similar bulk and density to NAC.

Medications are over-encapsulated to maintain the double-blind and participants are instructed to take two capsules in the morning and evening. At the end of the 12-week treatment period, the participant, clinician and the researcher are asked to record the drug the participant is thought to have received. Limiting the dose is permitted according to clinician judgement and is recorded in order to optimise treatment outcomes by minimising side effects and maximising adherence. Any participants who wish ongoing treatment are offered open-label pharmacotherapy at their own expense subject to the judgement of their clinicians. Clinical data for any such individuals are being collected.

### Medical management

At each treatment visit, all participants receive 20–30 min of medical management (MM), which was developed for the COMBINE Trial as a medically oriented intervention to maximise medication adherence in the treatment of AUD.^35^ Clinicians with minimal specialty training who are knowledgeable about AUD can deliver this brief, effective and widely used intervention. MM is thus feasible in all study sites and unlikely to obscure a medication effect on drinking outcomes. In MM, the clinician highlights the participant’s symptoms related to problematic alcohol use and the need for treatment. The participant is advised to reduce or stop drinking, is educated briefly about alcohol-related disorders, is provided a rationale to take medication, is instructed on the importance of medication adherence and is given information on the medications. Participants are not permitted to commence concurrent psychotherapy outside of the trial or, if necessary, they will be encouraged to defer commencement until at least the second half of the trial. Previous trials indicate this number to be low but nonetheless, such cases will be included as covariates in sensitivity analyses. The research nurse also checks the participant’s breath alcohol level, vital signs and weight, medication adherence, drinking and smoking, and general functioning and obtains a urine sample for measure of medication adherence. Participants provide reports of side effects at each study visit. Participants are asked to return unused study medication at each treatment visit for capsule counts and usage is recorded.

### Procedures and schedule of visits

Participants are initially assessed by a medical officer. If clinically indicated, participants are offered withdrawal management. Eligible participants are then offered further screening. The schedule of enrolment, interventions and assessments is depicted in table 1.

**Table 1:** The schedule of enrolment, interventions and assessments

Visit		Visit 1a—screening	Visit 1b—baseline	Visit 2	Visit 3	Visit 4	Visit 5	Visit 6—EOT	Visit 7—FU
**Time from baseline**		–	**0**	**Week 1**	**Week 3**	**Week 6**	**Week 9**	**Week 12**	**Week 24**
Informed consent & assessment of eligibility		X							
Pregnancy test—urine (if applicable)		X							
Medical examination		X						X	X
Demographics			X						
Questionnaires	SCID-5-RV, MoCA,	X							
	SAPAS		X						
	FTND		X						
	ADS, DrInC-2L		X					X	X
	BITe, CIS, EQ-5D-5L, HSUQ, IPAQ-Long, PCL-5, SCWT, SF-36, SIDAS, TMT		X					X	X
	ACEQ, AHS, DASS, ISI, TLFB (alcohol & smoking)		X	X	X	X	X	X	X
	DrInC-2R							X	X
Medical management—incl. AEs			X	X	X	X	X	X	X
Medication dispense			X		X	X			
Medication adherence				X	X	X	X	X	
Blood sampling (PEth, LFTs, GPxBC, GSHBC)			X			X		X	X

Time between screening and baseline testing <7 days.

ACEQ, Alcohol Craving Experience Questionnaire; ADS, Alcohol Dependence Scale; AEs, adverse events; AHS, Alcohol Hangover Scale; BITe, Brief Irritability Test; CIS, Cognitive Impulsivity Suite; DASS, Depression, Anxiety & Stress Scale; DrInC-2R/2L, Drinker Inventory of Consequences – Recent/Lifetime; EOT, End of Treatment; EQ-5D-5L, EuroQol Health-Related Quality of Life Questionnaire 5-Level; FTND, Fagerstrom Test for Nicotine; FU, Follow Up; GPxBC, Glutathione Peroxidase Blood Cells; GSHBC, Glutathione Blood Cells; HSUQ, Health Service Use Questionnaire; IPAQ-Long, International Physical Activity Questionnaire – Long; ISI, Insomnia Severity Index; LFTs, Liver Function Tests; MoCA, Montreal Cognitive Assessment; PCL-5, PTSD Checklist for DSM-5; PEth, phosphatidylethanol; SAPAS, Standardised Assessment of Personality Abbreviated Scale; SCID-5-RV, Structured Clinical Interview for DSM-5 – Research Version; SCWT, Stroop Color and Word Test; SF-36, Short Form 36; SIDAS, Suicidal Ideation Attributes Scale; TLFB, Timeline Follow-Back; TMT, Trail Making Test.

*Visit 1a (screening visit):* At the screening visit (visit 1), participants are asked to provide informed consent by the research nurse (see online supplemental Material). The research nurse obtains a structured medical and psychiatric history. Blood and urine samples are taken for clinical laboratory evaluations (see below).

*Visit 1b (baseline visit):* After screening (<7 days), during which time blood sampling and, if necessary, alcohol withdrawal management are completed, eligible participants undergo a research evaluation (90–120 min). A study physician performs a physical examination.

*Visits 1b-7 (treatment and follow-up visits):* Participants receive initial medical management and study medication during the first treatment session on the same day as the baseline visit. Participants are given either NAC or PL. During the 12-week treatment period, participants return for medical management and medication dispensing at weeks 1, 3, 6 and 9. They return for the final follow-up medical review and research appointments at week 12 and 24.

### Study assessments

*Medical/laboratory tests:* These tests screen participants for exclusion criteria, assess potential adverse effects, provide objective measures of alcohol use and assess covariates and secondary outcome measures. The physical examination at baseline includes blood pressure and cardiovascular examination, signs of alcohol-related liver disease, weight and height, a brief neurological examination and a mental state examination. Blood and urine are obtained at screening and at weeks 6, 12 and 24. Laboratory tests include urinalysis, urine toxicology, full blood count, liver function tests (LFTs: bilirubin, gamma-glutamyl transferase, alkaline phosphatase, aspartate aminotransferase, alanine aminotransferase, albumin, protein), coagulation tests, creatinine, redox markers (GPx blood cells, GSH blood cells) and phosphatidylethanol (PEth). All abnormal findings are reviewed by the physician and participation is endorsed or further evaluation performed as clinically indicated. Blood samples are also collected and stored for exploratory analyses relating to the current trial and for future use in ancillary studies.

*Questionnaires and tasks:* At baseline, demographics, medical history, personal and family history of AUD, and alcohol treatment history are obtained as in our previous trials.^36^,^39^ Psychiatric diagnostic information is obtained with the SCID-5-RV.^40^

The following measures are implemented at baseline and follow-up (see table 1):

Characteristics of alcohol use disorder (baseline and follow-up): (i) recent (last 30 days) alcohol consumption (frequency/quantity) assessed by the Timeline Follow-Back method (TLFB).^41^ We also measure tobacco use using the TLFB; (ii) severity of alcohol dependence assessed by the Alcohol Dependence Scale (ADS);^42^ (iii) craving for alcohol measured by the Alcohol Craving Experience Questionnaire (ACEQ);^43^ (iv) the Drinker Inventory of Consequences (DrInC-L: baseline; R: follow-up)^44^ provides a measure of the social, physical and emotional consequences of alcohol use; and (v) Fagerstrom Test for Nicotine Dependence^45^ to measure nicotine dependence.

Mental health: (i) the Depression, Anxiety & Stress Scale (DASS) measures the severity of symptoms of depression, anxiety and stress;^46^ (ii) sleep problems are assessed by the Insomnia Severity Index;^47^ (iii) Post Traumatic Stress Disorder (PTSD) checklist for DSM-5 (PCL,^48^) is used to measure symptoms of PTSD; (iv) Brief Irritability Test^49^ is used to measure irritability (BITe); (v) Suicidal Ideation Attributes Scale (SIDIS) is used to measure suicidal ideation^50^ ; (iv) Standardised Assessment of Personality Abbreviated Scale (SAPAS) is used to screen for personality disorders.^51^

Cognitive and executive function: (i) The Montreal Cognitive Assessment (MoCA) is used to screen for cognitive impairment (<24/30 excluded) that is likely to preclude the completion of study procedures and treatment;^52^ (ii) The Trail Making Test^53^ is used to measure the speed of processing and executive functioning; (iii) The Stroop Color and Word Test (SCWT) is used to measure executive functioning and capacity to inhibit cognitive interference.^54^

Physical health*:* (i) Health outcomes are measured using the EuroQol-5 Dimension (EQ-5D-5L) questionnaire^55^ and Short Form 36 (SF36^56^); (ii) Health and Service Use Questionnaire (HSUQ); and (iii) International Physical Activity Questionnaire – Long (IPAQ-L^57^).

We also collect daily drinking, medication usage and hangover symptoms once daily (adapted from the Alcohol Hangover Scale (AHS,^58^) via Smartphone Ecological Momentary Assessment) (SEMA3).

Assertive follow-up is employed to obtain data for all randomised participants regardless of whether they discontinue study medication and study visits. Follow-up data may be obtained by telephone in such cases.

*Side-effects monitoring:* Safety will be analysed using categorical outcomes, defined by the type and severity of adverse effects. Participants will provide side effects reports at each visit using the Systematic Assessment for Treatment Emergent Effects (SAFTEE) interview,^59^ a widely used measure of adverse events^60^ and for which a standardised approach exists for alcohol trials.^61^ Summary measures will then be developed.

### Data and Safety Monitoring

Adverse events are collected at every study visit and the site investigator rates their relationship to medication. Serious adverse events are defined as by the Therapeutics Goods Administration (TGA).^62^ If serious or unexpected adverse events occur during the trial, they are reviewed by the principal investigator and the trial team and reported within the specified time frames to the lead hospital ethics review committee (ERC) and to the Data and Safety Monitoring Board (DSMB). The DSMB comprises three members independent of the research study and funding body and meets regularly to provide oversight and monitoring to ensure participant safety and trial validity and integrity. The DSMB can request that the trial be terminated by the principal investigator in the event of safety concerns. Participants are assigned a code and their information de-identified in password-protected documents.

### Outcomes

*Primary outcome:* The primary outcome is alcohol consumption expressed as self-reported number of heavy drinking days per week, defined as >four drinks for women, >five drinks for men (where a single standard drink is defined by 10 g of ethanol). Any conflicting information will be validated by collateral reports and measurement of biological markers of alcohol consumption (PEth).

*Secondary outcomes:* Secondary alcohol outcomes include number of standard drinks per drinking day (SDDD) and absence of HDDs. We will also compare NAC vs PL on craving (ACEQ), alcohol dependence severity (ADS). Other secondary outcomes include adverse events, markers of liver injury (LFTs), levels of anxiety and depression (DASS), suicidal ideation (SIDAS), hangover symptoms (AHS), sleep disturbances (ISI) and cognitive functioning. We will explore the degree to which these clinical characteristics moderate treatment response and the mechanisms underlying these phenomena.

### Data analysis

*Sample size calculations:* A total of 180 AUD participants will be recruited for this study. We consider power for the primary outcome measure, HDD per week (analysed over 12 weeks). We regard Poisson means as approximately normally distributed, with variances equal to the means, adapting the methods of Hedeker *et al*.^63^ We consider power for a two-sided test at α=0.05, with n=180. We assume a within-subject correlation of 0.4, based on prior studies.^7^ For the effect of NAC vs placebo (treatment × time), we have >80% power to detect a small to medium effect where the groups separate to a small to medium effect (d=0.3) by week 12.

*Statistical analysis:* All available data from participants who took at least one dose of medication will be used to follow intention-to-treat principles. The primary outcome measure is frequency of HDDs (defined above), a sensitive, clinically relevant measure in alcohol trials.^64^ The two groups will first be compared for repeated measures of HDD frequency/week (weekly) using mixed effects models for count responses. Models can also be extended to allow for zero inflation, if patterns of abstinence yield more zero counts of HDDs than can be accommodated by a Poisson model. Secondary outcomes will also be examined using mixed models. Additional covariates and predictors will be included for sensitivity analyses. Categorical variables will be compared using a χ² test. Participants will be followed irrespective of whether they continue to receive treatment. As with all alcohol treatment studies, we anticipate some participant dropout and intermittent missing data which will be estimated through multiple imputation and assessed via sensitivity analysis.

*Cost-effectiveness analysis (CEA*): A cost-efficacy analysis will be conducted from both health sector and societal perspectives. As per our previous work, the primary outcome will be Quality Adjusted Life Years calculated from the Short Form 36. Participants will be consented for access to data on healthcare service use through Medicare and Pharmaceutical Benefits Schedules. A brief Health Services Use Questionnaire will capture additional resource use such as hospitalisations and lost productivity. Standardised economic evaluation techniques including incremental analysis of mean differences and bootstrapping to determine confidence intervals will be used. If the intervention is cost-efficacious, modelling techniques will be used to estimate the cost-effectiveness of NAC for the AUD population across Australia.

### Ethics and Dissemination

Ethics approval for the study has been granted by The Sydney Local Health District Ethics Review Committee (X21-0342& HREC2021/ETH11614). Participants are provided with an approved plain language information sheet and provide written content to participate. The protocol is version 8. The study involves off-label use of a registered medication in Australia such that approval for this trial has been given under the Clinical Trial Notification (CTN) scheme of the TGA. The trial is sponsored by Sydney Local Health District and was registered with the NIH clinical trials registry on 7 June 2022 (NCT05408247). Participants are advised of their right to seek compensation from the study sponsor in the event of harm arising from trial participation. Protocol amendments to the clinical protocol will be updated to the clinical trials registry following approval by the corresponding ethics review committee. The Consumer Advisory Group and trial participants will be kept informed about study process, the final results and a lay summary provided to study sites for distribution. The trial is now in the recruitment phase (expected completion: 2026). There are no restrictions on publication from the sponsor or other parties. Publications will be authored by the investigators and authorship offered according to NHMRC criteria. De-identified data will be available at request from the investigators.

### Patient and Public Involvement

The NAC-AUD study team have extensive experience engaging with the community, having maintained active involvement with consumers including regular feedback of results from clinical trials and ongoing feedback regarding design and implementation of research protocols (via Edith Collins Consumer Advisory Group, Royal Prince Alfred Hospital; Drug and Alcohol Clinical Research and Improvement Network Consumer Advisory Group). Co-design with consumers included: appointment schedule, dosing scheduling and tolerability, examining key factors leading to relapse, including relevant outcomes such as reduction in heavy drinking and quality of life rather than a focus on survival time to lapse (abstinence). This also aligns with the current evidence base.^65^ The Consumer Advisory Group and trial participants will be kept informed about study process, the final results and a lay summary provided to study sites for distribution.

## Supplementary material

10.1136/bmjopen-2024-091631 online supplemental file 1

## References

[1] World Health Organization (2019). Global status report on alcohol and health 2018.

[2] Haber PS, Riordan BC, Winter DT (2021). New Australian guidelines for the treatment of alcohol problems: an overview of recommendations. Medical Journal of Australia.

[3] Morley KC, Perry CJ, Watt J (2021). New approved and emerging pharmacological approaches to alcohol use disorder: a review of clinical studies. Expert Opin Pharmacother.

[4] Morley KC (2021). Commentary on Donoghue: Low prescribing rates of pharmacotherapy for alcohol use disorder limit potential public health impact. Addiction.

[5] Stocklosa T, Morley KC, Volovets A (2018). Pharmacotherapy for alcohol use disorder and alcoholic liver disease. Curr Addict Rep.

[6] Agabio R, Sinclair JM, Addolorato G (2018). Baclofen for the treatment of alcohol use disorder: the Cagliari Statement. Lancet Psychiatry.

[7] Morley KC, Baillie A, Fraser I (2018). Baclofen in the treatment of alcohol dependence with or without liver disease: multisite, randomised, double-blind, placebo-controlled trial. Br J Psychiatry.

[8] Morley KC, Kranzler HR, Luquin N (2024). Topiramate Versus Naltrexone for Alcohol Use Disorder: A Genotype-Stratified Double-Blind Randomized Controlled Trial. Am J Psychiatry.

[9] Morley KC, Baillie A, Van Den Brink W (2018). N-acetyl cysteine in the treatment of alcohol use disorder in patients with liver disease: Rationale for further research. Expert Opin Investig Drugs.

[10] Schmitt B, Vicenzi M, Garrel C (2015). Effects of N-acetylcysteine, oral glutathione (GSH) and a novel sublingual form of GSH on oxidative stress markers: A comparative crossover study. Redox Biol.

[11] Quintanilla ME, Morales P, Ezquer F (2018). Commonality of Ethanol and Nicotine Reinforcement and Relapse in Wistar-Derived UChB Rats: Inhibition by N-Acetylcysteine. Alcohol Clin Exp Res.

[12] Adams C, Conigrave JH, Lewohl J (2020). Alcohol use disorder and circulating cytokines: A systematic review and meta-analysis. Brain Behav Immun.

[13] Adams C, Perry N, Conigrave J (2023). Central markers of neuroinflammation in alcohol use disorder: A meta-analysis of neuroimaging, cerebral spinal fluid, and postmortem studies. *Alcohol Clin Exp Res (Hoboken*).

[14] Yang M, Zhou X, Tan X (2022). The Status of Oxidative Stress in Patients with Alcohol Dependence: A Meta-Analysis. Antioxidants (Basel).

[15] Morley KC, Lagopoulos J, Logge W (2018). Neurometabolite Levels in Alcohol Use Disorder Patients During Baclofen Treatment and Prediction of Relapse to Heavy Drinking. Front Psychiatry.

[16] Hwa L, Besheer J, Kash T (2017). Glutamate plasticity woven through the progression to alcohol use disorder: a multi-circuit perspective. F1000Res.

[17] Lebourgeois S, González-Marín MC, Jeanblanc J (2018). Effect of N-acetylcysteine on motivation, seeking and relapse to ethanol self-administration. Addict Biol.

[18] Quintanilla ME, Rivera-Meza M, Berríos-Cárcamo P (2016). Beyond the “First Hit”: Marked Inhibition by N-Acetyl Cysteine of Chronic Ethanol Intake But Not of Early Ethanol Intake. Parallel Effects on Ethanol-Induced Saccharin Motivation. Alcohol Clin Exp Res.

[19] Schneider R, Bandiera S, Souza DG (2017). N-acetylcysteine Prevents Alcohol Related Neuroinflammation in Rats. Neurochem Res.

[20] Bradlow RCJ, Berk M, Kalivas PW (2022). The Potential of N-Acetyl-L-Cysteine (NAC) in the Treatment of Psychiatric Disorders. CNS Drugs.

[21] Grant JE, Odlaug BL, Chamberlain SR (2014). A randomized, placebo-controlled trial of N-acetylcysteine plus imaginal desensitization for nicotine-dependent pathological gamblers. J Clin Psychiatry.

[22] Grant JE, Chamberlain SR, Redden SA (2016). N-Acetylcysteine in the Treatment of Excoriation Disorder: A Randomized Clinical Trial. JAMA Psychiatry.

[23] Prado E, Maes M, Piccoli LG (2015). N-acetylcysteine for therapy-resistant tobacco use disorder: a pilot study. Redox Rep.

[24] Schulte MHJ, Wiers RW, Boendermaker WJ (2018). The effect of N-acetylcysteine and working memory training on cocaine use, craving and inhibition in regular cocaine users: correspondence of lab assessments and Ecological Momentary Assessment. Addict Behav.

[25] McKetin R, Dean OM, Turner A (2021). N-acetylcysteine (NAC) for methamphetamine dependence: A randomised controlled trial. EClinicalMedicine.

[26] Padoei F, Mamsharifi P, Hazegh P (2023). The therapeutic effect of N-acetylcysteine as an add-on to methadone maintenance therapy medication in outpatients with substance use disorders: A randomized, double-blind, placebo-controlled clinical trial. Brain Behav.

[27] Chang CT, Hsieh PJ, Lee HC (2021). Effectiveness of N-acetylcysteine in Treating Clinical Symptoms of Substance Abuse and Dependence: A Meta-analysis of Randomized Controlled Trials. Clin Psychopharmacol Neurosci.

[28] Coppersmith V, Hudgins S, Stoltzfus J (2021). The use of N-acetylcysteine in the prevention of hangover: a randomized trial. Sci Rep.

[29] Squeglia LM, Tomko RL, Baker NL (2018). The effect of N-acetylcysteine on alcohol use during a cannabis cessation trial. Drug Alcohol Depend.

[30] Morley KC, Peruch S, Adams C (2023). N acetylcysteine in the treatment of alcohol use disorder: a randomized, double-blind, placebo-controlled trial. Alcohol Alcohol.

[31] Li T, Boutron I, Salman RA-S (2017). Review and publication of protocol submissions to Trials – what have we learned in 10 years?. Trials.

[32] Chan A-W, Tetzlaff JM, Altman DG (2013). SPIRIT 2013 Statement: Defining standard protocol items for clinical trials. Ann Intern Med.

[33] Anton RF, Litten RZ, Falk DE (2012). The Alcohol Clinical Trials Initiative (ACTIVE): Purpose and Goals for Assessing Important and Salient Issues for Medications Development in Alcohol Use Disorders. Neuropsychopharmacol.

[34] Morley KC, Teesson M, Sannibale C (2009). Sample bias from different recruitment strategies in a randomised controlled trial for alcohol dependence. Drug Alcohol Rev.

[35] Pettinati H, Donovan D, Ernst D (2004). Medical Management Treatment Manual: A Clinical Research Guide for Medically Trained Clinicians Providing Pharmacotherapy as Part of the Treatment for Alcohol Dependence.

[36] Morley KC, Leung S, Baillie A (2013). The efficacy and biobehavioural basis of baclofen in the treatment of alcoholic liver disease (BacALD): study protocol for a randomised controlled trial. Contemp Clin Trials.

[37] Morley KC, Teesson M, Reid SC (2006). Naltrexone versus acamprosate in the treatment of alcohol dependence: A multi-centre, randomized, double-blind, placebo-controlled trial. Addiction.

[38] Morley KC, Baillie A, Leung S (2014). Baclofen for the Treatment of Alcohol Dependence and Possible Role of Comorbid Anxiety. *Alcohol Alcohol*.

[39] Morley KC, Baillie A, Sannibale C (2013). Integrated care for comorbid alcohol dependence and anxiety and/or depressive disorder: study protocol for an assessor-blind, randomized controlled trial. Addict Sci Clin Pract.

[40] First MB, Williams JBW, Karg RS (2015). Structured Clinical Interview for DSM5—Research Version (SCID-5 for DSM-5, Research Version; SCID-5-RV).

[41] Sobell LC, Sobell MB, Leo GI (1988). Reliability of a Timeline Method: assessing normal drinkers’ reports of recent drinking and a comparative evaluation across several populations. Br J Addict.

[42] Skinner HA, Allen BA (1982). Alcohol dependence syndrome: measurement and validation. J Abnorm Psychol.

[43] Coates JM, Gullo MJ, Feeney GFX (2017). The Mini Alcohol Craving Experience Questionnaire: Development and Clinical Application. Alcohol Clin Exp Res.

[44] Miller WR, TJ S, Longabaugh R (1995). The Drinker Inventory of Consequences (DrInC): An Instrument for Assessing Adverse Consequences of Alcohol Abuse.

[45] Heatherton TF, Kozlowski LT, Frecker RC (1991). The Fagerström Test for Nicotine Dependence: a revision of the Fagerström Tolerance Questionnaire. Br J Addict.

[46] Lovibond PF, Lovibond SH (1995). The structure of negative emotional states: Comparison of the Depression Anxiety Stress Scales (DASS) with the Beck Depression and Anxiety Inventories. Behav Res Ther.

[47] Bastien CH, Vallières A, Morin CM (2001). Validation of the Insomnia Severity Index as an outcome measure for insomnia research. Sleep Med.

[48] Weathers FW, Blake DD, Schnurr PP (2013). Clinician-Administered PTSD Scale for DSM-5 (CAPS-5).

[49] Holtzman S, O’Connor BP, Barata PC (2015). The Brief Irritability Test (BITe): a measure of irritability for use among men and women. Assessment.

[50] van Spijker BAJ, Batterham PJ, Calear AL (2014). The Suicidal Ideation Attributes Scale (SIDAS): Community‐Based Validation Study of a New Scale for the Measurement of Suicidal Ideation. Suicide & Life Threat Behav.

[51] Hesse M, Moran P (2010). Screening for personality disorder with the Standardised Assessment of Personality: Abbreviated Scale (SAPAS): further evidence of concurrent validity. BMC Psychiatry.

[52] Nasreddine ZS, Phillips NA, Bédirian V (2005). The Montreal Cognitive Assessment, MoCA: a brief screening tool for mild cognitive impairment. J Am Geriatr Soc.

[53] Mitrushina MN, Boone KL, D’Elia L (1999). Handbook of Normative Data for Neuropsychological Assessment.

[54] Stroop JR (1935). Studies of interference in serial verbal reactions. J Exp Psychol.

[55] Balestroni G, Bertolotti G (2012). EuroQol-5D (EQ-5D): an instrument for measuring quality of life. Monaldi Arch Chest Dis.

[56] Brazier JE, Harper R, Jones NM (1992). Validating the SF-36 health survey questionnaire: new outcome measure for primary care. BMJ.

[57] Craig CL, Marshall AL, Sjöström M (2003). International physical activity questionnaire: 12-country reliability and validity. Med Sci Sports Exerc.

[58] Rohsenow DJ, Howland J, Minsky SJ (2007). The Acute Hangover Scale: A new measure of immediate hangover symptoms. Addict Behav.

[59] Levine J SN (1986). SAFTEE: a technique for the systematic assessment of side effects in clinical trials. Psychopharmacol Bull.

[60] Anton RF, O’Malley SS, Ciraulo DA (2006). Combined pharmacotherapies and behavioral interventions for alcohol dependence: the COMBINE study: a randomized controlled trial. JAMA.

[61] Johnson BA, Ait-Daoud N, Roache JD (2005). The COMBINE SAFTEE: a structured instrument for collecting adverse events adapted for clinical studies in the alcoholism field. J Stud Alcohol Suppl.

[62] (2017). Pharmacovigilance responsibilities.

[63] Hedeker D, Gibbons RD, Waternaux C (1999). Sample size estimation for longitudinal designs with attrition. Journal of Educational and Behavoral Statistics.

[64] Falk D, Wang XQ, Liu L (2010). Percentage of Subjects With No Heavy Drinking Days: Evaluation as an Efficacy Endpoint for AlcoholClinical Trials. Alcoholism Clin &amp; Exp Res.

[65] Witkiewitz K, Falk DE, Litten RZ (2019). Maintenance of World Health Organization Risk Drinking Level Reductions and Posttreatment Functioning Following a Large Alcohol Use Disorder Clinical Trial. *Alcoholism Clin &amp; Exp Res*.

