# Supplementary material for: N-acetyl cysteine for the treatment of alcohol use disorder: study protocol for a multi-site, double-blind randomised controlled trial (NAC-AUD study)
Source: BMJ Open. 2025 Sep 8;15(9):e091631. doi: 10.1136/bmjopen-2024-091631 (PMC12421191; doi:10.1136/bmjopen-2024-091631)
Supplement: online supplemental file 1 [file bmjopen-15-9-s001.docx]

**Participant Information Sheet & Consent Form**

**Interventional Study** – *Adult providing own consent*

| **Study Name** | A Randomised Controlled Trial of N-Acetylcysteine for the Management of Alcohol Use Disorder |
| --- | --- |
| **Short Title** | NAC-AUD |
| **Protocol No.:** | X21-0342 |
| **HREC No.** | 2021/ETH11614 |
| **Local Ref No.** | *INSERT LOCAL REFERENCE NUMBER* |
| **Sponsor** | The University of Sydney |
| **Chief Investigator** | Prof Kirsten Morley |
| **Local Principal Investigator** | *INSERT SITE PI NAME* |

# INTRODUCTION

You are invited to take part in a research study which will investigate whether N-acetylcysteine (NAC) is a safe and effective treatment for reducing heavy alcohol consumption. While there is evidence to show that NAC may be helpful to reduce drinking and craving in individuals with Alcohol Use Disorder (AUD), there is little understanding as to how this may occur.

Medications, drugs and devices have to be approved for use by the Australian Federal Government. NAC has been used safely in the treatment of Chronic Obstructive Pulmonary Disease (COPD) for many years and new evidence suggests it may also help reduce drinking and craving in alcohol-dependent individuals. This study will be conducted under the Therapeutic Goods Administration Clinical Trials Notification Scheme. This allows the investigators to use this product for medical research purposes once the research has been assessed and approved by an authorised Human Research Ethics Committee (HREC).

You have been invited to participate in this study because you have expressed a desire to reduce your alcohol intake.

Please read this information carefully. Ask questions about anything that you don’t understand or want to know more about. Before deciding whether or not to take part in this study, you might want to talk about it with a relative, friend, or your doctor. If English is not your first language and you require an interpreter, one will be provided for you.

Your participation in this study is completely voluntary and there will be no cost to you. If you do not want to take part in this study you do not have to. You should feel under no obligation to participate in this study. Choosing not to take part in this study will not affect your current and future medical care in any way.

If you decide you want to take part in the research project, you will be asked to sign the consent section. By signing it you are telling us that you:

- Understand what you have read.
- Consent to take part in the research project.
- Consent to have the tests and treatments that are described.
- Consent to the use of your personal and health information as described.

Additionally, you will be asked to sign a separate consent form authorising the study to access your Services Australia information, see the separate Services Australia Participant Information Document and Participant Consent Form. Services Australia is not involved in this research other than to provide the information that you have consented to the release of, should you decide to participate in this study. Services Australia has confirmed that this research and any associated documents have received approval from a Human Research Ethics Committee (HREC) that is registered with and operates within guidelines set out by the National Health and Medical Research Council (NHMRC).

You will be given a copy of this Participant Information Sheet and Consent Form to keep.

# DESCRIPTION OF THE STUDY

This is a randomised, double-blind, controlled trial. Randomised means that you will be randomly assigned to one of two treatment groups: NAC or matched placebo (an identical capsule containing no active drug). This is similar to flipping a coin, but will be done by a computer. Double-blind means that neither you, nor the study staff will know in advance the treatment group you are in. In case of medical need/emergency, your treatment group can be quickly identified. You can choose to be notified of the treatment you received during the study at the end of the entire trial, rather than at the end of your participation in the trial.

# WHAT THIS STUDY INVOLVES

You will be asked to take 4 capsules daily – two in the morning, and two in the evening. This is a total of 2400mg of NAC or 2400mg placebo. You will continue with this daily treatment for a period of 12 weeks.

During the treatment period you will need to attend the clinic at the following time points for the listed assessments:

- Screening & Baseline – Week 0
- On-Treatment Visits – Weeks 1, 3, 6, 9
- End of Treatment – Week 12
- Follow-Up – Week 24 – *this visit may be completed over the phone*

# STUDY PROCEDURES

## Screening & Baseline Visit – 90 Minutes

If you decide to participate in this study, you will be asked to sign the consent form below before any assessments are performed. You will be asked to undergo an initial medical assessment, conducted by a study doctor, in order to check whether or not the study is suitable for you. These tests will include an evaluation of your medical and alcohol use history including drug use history, other medications you use, mental health history, and trauma history. Demographic data (including ethnicity, age, sex and diet) will be recorded as well as your height, weight, blood pressure and heart rate. The researchers would like to have access to your medical record to obtain information relevant to this study. If appropriate, a pregnancy test (urine test) will be performed to confirm you’re not pregnant. A blood sample (approx. 20-30 mLs) will also be collected for routine screening and for research purposes. You will also be asked to complete a number of questionnaires about your alcohol consumption, nicotine dependence, craving for alcohol, and mood.

If you are eligible to participate in the study, you will be randomised once the results of your blood tests are known. You will be given 3 weeks of medication and instructed to take 2 capsules in the morning and 2 in the evening. If you miss a dose and it has been less than 6 hours since your usual dose time, take the dose. If it has been more than 6 hours, skip the dose and take your next scheduled dose. Do not take a double dose if you have missed a dose of your medication.

If you experience significant alcohol withdrawal symptoms, you will be offered additional treatment and will be able to join the study once these symptoms have resolved.

## On-Treatment Visits (Weeks 1, 3, 6, & 9) – 60 minutes

Each on-treatment visit will include medical management, in which the study team will ask you about how you’re going with the study and any side effects, or other medications you’re taking. You will be asked to complete a number of questionnaires about your alcohol consumption, craving for alcohol, nicotine dependence mood and sleep. The study team will count your medication at each visit and dispense new medication at Week 3 and Week 6. You will also be asked to provide a blood sample at Week 6 (approx. 15-20mL).

## End of Treatment Visit (Week 12) & Follow-up Visit (Week 24) – 60 minutes

These visits will include medical management, in which the study team will ask you about how you’re going with the study and any side effects, or other medications you’re taking. You will be asked to complete a number of questionnaires about your alcohol consumption, craving for alcohol, nicotine dependence, mood and sleep. At Week 12 the study team will count and collect your medication, you will be asked to provide a blood sample (approx. 10-20mL) and you will be weighed again. A routine medical assessment will be conducted by a study doctor at week 12 and 24 (if visit is in clinic).

# RISKS

All medical procedures – whether for diagnosis or treatment, routine or experimental – involve some risk of injury. In addition, there may be risks associated with this study that are presently unknown and unforeseeable. Despite all safety precautions, you may develop medical complications from participating in this study.

## Side Effects from NAC

NAC has been tested in a variety of populations and is generally well tolerated at moderate doses (600-1200mg twice daily) with mild side effects. Gastrointestinal events (i.e. heartburn, flatulence, mild abdominal cramps) are the most commonly reported side effects and are generally temporary and resolved over time. Other less common reported adverse effects include pruritus (itching), headache, flatulence/diarrhoea, abdominal cramps, local rash, fatigue, increased blood pressure, sweating, ears popping, increased appetite, canker sores, chest pain, crying, and dizziness. None have been serious, and it has been well documented that NAC is safe and well-tolerated among study participants. Allergic reactions to NAC have occurred and seizures have been reported in rare cases, but only at very high intravenous doses. In this study, you will be dispensed oral capsules at much lower doses.

You are advised not to drive or operate machinery until you are certain about how the medication affects you.

## Blood Sampling

Blood collection involves some discomfort at the site from which the blood is taken. There is also a risk of some minor bruising at the site, which may last one to two days.

## For People of Child-Bearing Capability

It is important that people of child-bearing capability participating in this study are not pregnant and do not become pregnant during the course of the study. The safety and efficacy of NAC in pregnant or breastfeeding people has not been established. If you are of child-bearing capability and there is any possibility that you are pregnant, the researchers will perform a pregnancy (urine) test before you start in the study. If necessary, you should use reliable contraception (such as implanted contraception, an IUD or have had a tubal ligation) while participating in the study. If at any time you think you may have become pregnant, it is important to let the researchers know immediately.

## Registry Consent

You will be asked to sign a consent form authorising the study to access your complete Medicare Benefits Schedule (MBS) and/or Pharmaceutical Benefits Scheme (PBS) and/or Australian Immunisation Register (AIR) data as outlined in the consent form. Medicare collects information on your doctor visits and the associated costs, while the PBS collects information on the prescription medications you have filled at pharmacies. The AIR is a national register that records vaccines given to people of all ages in Australia. The consent form is sent securely to Services Australia who holds MBS, PBS and AIR data confidentially.

# BENEFITS

While we intend that this research study furthers medical knowledge and may improve treatment of alcohol dependence in the future, it may not be of direct benefit to you.

# COMPENSATION FOR INJURIES OR COMPLICATIONS

If you suffer any injuries or complications as a result of this study, you should contact the study doctor as soon as possible, who will assist you in arranging appropriate medical treatment. If you are eligible for Medicare, you can receive any medical treatment required to treat the injury or complication, free of charge, as a public patient in any Australian public hospital.

In addition, you may have a right to take legal action to obtain compensation for any injuries or complications resulting from the study. Compensation may be available if your injury or complication is sufficiently serious and is caused by unsafe drugs or equipment, or by the negligence of one of the parties involved in the study (for example, the researcher, the hospital, or the treating doctor). You do not give up any legal rights to compensation by participating in this study.

# COSTS

Participation in this study will not cost you anything, nor will you be paid. Each participant will be reimbursed for out-of-pocket expenses, inconvenience and time involved. Such reimbursement is standard practice in studies such as this. Participants will be reimbursed $30 for attendance to the End of Treatment Visit (Week 12) and Follow-up Visit (Week 24). This will be provided in the form of shopping vouchers for non-alcoholic goods.

# VOLUNTARY PARTICIPATION

Participation in this study is entirely voluntary. You do not have to take part in it. If you do take part, you can withdraw at any time without having to give a reason. Whatever your decision, please be assured that it will not affect your medical treatment or your relationship with the staff who are caring for you. Sometimes during the course of a study, new information becomes available about the treatment that is being studied. While you are participating in this study, you will be kept informed of any significant new findings which may affect your willingness to continue in the study.

You can withdraw from the study at any time by completing and signing the ‘Participant Withdrawal of Consent Form’, which will be given to you if you decide to withdraw. If you withdraw from the study you will be able to choose whether the study will destroy or retain the information it has collected about you. You should only choose one of these options. Where both boxes are ticked in error or neither box is ticked, the study will destroy all information it has collected about you. If you authorised the study to access your Services Australia information, then you will also need to complete and sign the ‘Services Australia Participant Withdrawal of Consent Form’.

# CONFIDENTIALITY

All data collected will be entered electronically and stored on a research database named REDCap (Research Electronic Data Capture). This is a secure, web-based, non-commercial, data management tool designed for research purposes, hosted and backed up on the University of Sydney’s servers on a daily basis. No personnel other than the researchers will have access to the research documents. The data will be analysed by the researchers at the University of Sydney. Your de-identified data may be used for future research projects. The researchers will seek prior ethics approval for any future use or collaboration of/with the data. All data for use in journal publications and presentations will be de-identified. The files will be retained for 15 years from the day the study is completed. Once this retention expires, the files will be disposed of using the University of Sydney’s confidential waste disposal service.

# WHAT WILL HAPPEN TO MY RESEARCH SAMPLES?

Blood samples that are collected for Phosphatidyl ethanol (PEth) will be couriered to the Biochemistry Department at the Royal Prince Alfred Hospital. Processing of PEth will occur immediately. All remaining samples will be stored at Drug Health Services at the Royal Prince Alfred Hospital for analysis at the end of the trial (i.e. for oxidative stress markers & genotyping purposes). If you withdraw from the study, samples stored for research purposes will not be destroyed as they will be stored without any identifiable data.

Blood samples that are taken to ensure you remain healthy during the course of the trial will be analysed at the study site then destroyed according to standard procedures. All samples will be destroyed after analysis in accordance with your site’s Standard Operating Procedures at study closure. Samples collected for potential genotyping will not be identifiable and will not be assessed in real time. These samples will not be reviewed for any clinical significance but rather for any genetic markers of treatment response. Full details on what these samples will be assessed for are unknown and will be determined on the basis of any new research and data in the field. Any findings will not be relayed back to you.

Urine samples are collected at the baseline visit to confirm that participants of child-bearing potential are not pregnant. Urine samples may also be collected if you think you have become pregnant during the study. Once determined, urine samples will be destroyed in accordance with the Standard Operating Procedures of the study site.

# FURTHER INFORMATION

When you have read this information, *INSERT PI NAME* or one of their associates will discuss it with you further and answer any questions you may have. If you would like to know more at any stage, please feel free to contact *INSERT PI NAME* on *INSERT PI CONTACT NUMBER*.

This Participant Information Sheet is for you to keep.

# ETHICS APPROVAL

This study has been approved by the Human Research Ethics Committee (RPAH Zone) of the Sydney Local Health District (SLHD). Any person with concerns or complaints about the conduct of this study should contact the **Executive Officer on 02 9515 6766** and quote **ethics approval number: 2021/ETH11614**.

The conduct of this study at *INSERT SITE NAME* has been authorised by the *INSERT GOVERNANCE NAME.* Any person with concerns or complaints about the conduct of this study may also contact the Research Governance Officer on *INSERT GOVERNANCE CONTACT NO.* and quote **local reference number:** *INSERT LOCAL REFERENCE NUMBER* *.*

**Consent Form**

**Interventional Study** – *Adult providing own consent*

| **Study Name** | A Randomised Controlled Trial of N-Acetylcysteine for the Management of Alcohol Use Disorder |
| --- | --- |
| **Short Title** | NAC-AUD |
| **Protocol No.:** | X21-0342 |
| **HREC No.** | 2021/ETH11614 |
| **Local Ref No.** | *INSERT LOCAL REFERENCE NUMBER* |
| **Sponsor** | The University of Sydney |
| **Chief Investigator** | Prof Kirsten Morley |
| **Local Principal Investigator** | *INSERT SITE PI NAME* |

I, ...................................................................................................................................... *[participant name]*

of..............................................................................................................................……. *[participant address]*

have read and understood the ‘Participant Information Sheet’ of the above-named research study.

I have discussed the study with ____________________________*INSERT INVESTIGATOR/DELEGATE NAME*

| I have been made aware of the procedures involved in the study, including any known or expected inconvenience, risk, discomfort or potential side effect and of their implications as far as they are currently known by the researchers. |
| --- |
|  |
| I understand that my participation in this study will allow the researchers to have access to my medical record, and I agree to this. |
|  |
| I understand that genotyping will be performed on a sample of my blood to test whether some variations in genes predict response to NAC and I agree to this. |
|  |
| I understand that my de-identified data may be used for future research and I agree to this. |
|  |
| I would like to receive a copy of the study results when they become available. My email address is:  ___________________________________ |
|  |
| I freely choose to participate in this study and understand that I can withdraw at any time. |
|  |
| I also understand that the research study is strictly confidential. |

I hereby agree to participate in this research study.

| **Participant Name** |  |  |  |
| --- | --- | --- | --- |
| **Signature** |  | **Date** |  |
|  |  |  |  |
| **Investigator Name** |  |  |  |
| **Signature** |  | **Date** |  |
|  |  |  |  |
