# Supplementary material for: N-acetyl cysteine for the treatment of alcohol use disorder: study protocol for a multi-site, double-blind randomised controlled trial (NAC-AUD study)
Source: BMJ Open. 2025 Sep 8;15(9):e091631. doi: 10.1136/bmjopen-2024-091631 (PMC12421191; doi:10.1136/bmjopen-2024-091631)
Supplement: Supplementary file 2 [file bmjopen-2024-091631.reviewer_comments.pdf]

PEER REVIEW HISTORY

BMJ Open publishes all reviews undertaken for accepted manuscripts. Reviewers are asked to complete a checklist review form and are provided with free text boxes to elaborate on their assessment. These free text comments are reproduced below.

ARTICLE DETAILS

Title (Provisional)

N-acetyl cysteine for the treatment of alcohol use disorder: study protocol for a multi-site, double-blind randomised controlled trial (NAC-AUD study)

Authors

Morley, Kirsten; Arunogiri, Shalini; Connor, Jason P; Clark, Paul J; Chatterton, Mary Lou; Baillie, Andrew; Slade, Tim; Berk, Michael; Lubman, D; Haber, Paul S

VERSION 1 - REVIEW

**Reviewer**	**1**
**Name**	**Kedia, Satish**
**Affiliation**	**The University of Memphis**
**Date**	**07-Oct-2024**
**COI**	**None**

N-acetyl cysteine for the treatment of alcohol use disorder: study protocol for a multi-site, double-blind randomized controlled trial (NAC-AUD study)

Thank you for the opportunity to review this interesting, proposed study. Generally, I found the study protocol to be well-conceived. It's findings could be impactful for many people struggling with AUD and those striving to provide effective treatment.

1. The introduction thoroughly describes the current state of evidence regarding medication for AUD and clearly lays out the study's hypotheses. I appreciate your thorough description of the randomization and blinding procedures, and also the thoughtful plans for patient safety and unblinding in case of medical emergency.

2. Page 8 of 33, Patient recruitment and sample details: Do you have demographic targets for your sample? For example, will you attempt to recruit equal numbers of men and women and a spread of ages? Are the demographic proportions of the treatment locations similar, or different? Will you account for gender, age, racial characteristics in the analysis?

3. Page 8 of 33, Patient recruitment and sample details: Is there a projected timeline for recruitment – a projected beginning and ending date? Will participants engage with the

program on a rolling basis, as clients are enrolled, or will the program commence and conclude all at once with a full roster of clients?

4. Page 11 of 33, line 52: I'm curious about the rationale for asking participants to defer concurrent psychotherapy for the first 6 weeks of the trial. Why 6 weeks? It's understandable to consider psychotherapy as an important modification to the treatment protocol, but I wonder how you will collect and analyze data around psychosocial and behavioral interventions in association with the prescription. I do not see it mentioned in the proposed analysis.

5. Page 16 of 33, lines 26-31: Is there a projected amount of drop out that you anticipate or a threshold that you are willing to accept?

6. The Discussion section is rather brief. It could be expanded to include alternative scenarios for the protocol, limitations, and research implications.

Findings could be impactful for many people struggling with AUD and those striving to provide effective treatment.

**Reviewer**	**2**
**Name**	**ghaderi, amir**
**Affiliation**	**Kashan University of Medical Sciences, addiction**
**Date**	**17-Mar-2025**
**COI**	**None**

The authors investigated Effects of "N-acetyl cysteine for the treatment of alcohol use disorder:

study protocol for a multi-site, double-blind randomized controlled trial (NAC-AUD study)". However, many points should be addressed in the revised version.

1. What has been the basis for the classification of the groups?

2. What is the justification in your choice of N-acetyl cysteine dose?

3. The keywords should be taken from the Medical Subject Headings.

4. What is the criterion for choosing an age range of 18 to 70 years? Specify. Do not have this problem at an older age.

5. Is there a precise documentation of side effects following international standards? Please provide this information.

6. Did not people study smoking and drug use? Do not use hypnotic drugs? Do you think these do not interfere with the study? How are they controlled?

7. No information about the physical activity records.

8. The table result is unclear. Please state the changes between the two groups in the variables examined, also β (95% CI).

9. In the discussion, refer to the new article published in this field." Padoei, Fateme, et al. "The therapeutic effect of N-acetylcysteine as an add-on to methadone maintenance therapy medication in outpatients with substance use disorders: A randomized, double-blind, placebo-controlled clinical trial." Brain and Behavior 13.1 (2023): e2823".

10. Please describe the statistical analyses in more detail. For example, did you adjust the outcome variable measured in the follow-up measurements for the baseline value of the outcome (according to the equation $Yt = \beta0 + \beta1^* X + \beta1^*Yt0$, where Yt = the outcome measured in the two follow-up measurements, X = treatment variable, β1 = overall treatment effect, and Yt0 = outcome variable measured in the baseline measurement)? Please indicate the parameters you have adjusted for in your linear regression analysis. It is well acceptable that an appropriate significance level α, such as 0.05, is pre-specified to guarantee the probability of incorrectly rejecting a single test of null hypothesis (H0) no larger than α. However, there are many situations where more than one or even a large number of hypotheses are simultaneously tested, which is referred to as multiple comparisons. Because you are testing many different hypothesis simultaneously ("multiple comparisons"), proper adjustment of statistical inference is required.

11. Did the drug (NAC) have side effects in obese adults?

12. Identify the primary and secondary outcomes.

VERSION 1 - AUTHOR RESPONSE

Reviewer: 1
N-acetyl cysteine for the treatment of alcohol use disorder: study protocol for a multi-site, double-blind randomized controlled trial (NAC-AUD study)
Thank you for the opportunity to review this interesting, proposed study. Generally, I found the study protocol to be well-conceived. It's findings could be impactful for many people struggling with AUD and those striving to provide effective treatment.

RC. The introduction thoroughly describes the current state of evidence regarding medication for AUD and clearly lays out the study's hypotheses. I appreciate your thorough description of the randomization and blinding procedures, and also the thoughtful plans for patient safety and unblinding in case of medical emergency.

AC: This is noted with thanks.

RC: Page 8 of 33, Patient recruitment and sample details: Do you have demographic targets for your sample? For example, will you attempt to recruit equal numbers of men and women and a spread of ages? Are the demographic proportions of the treatment

locations similar, or different? Will you account for gender, age, racial characteristics in the analysis?

AC: Given our extensive experience conducting clinical trials in this population we envisage that the sex ratio will be between 40-60% for males and females and average age of 45-50. In our Randomisation paragraph in the Methods section (last paragraph, page 7) we state that we will stratify for sex. We can conduct a secondary analysis of the role of age in our analysis but our experience in this field, which is consistent with the literature on alcohol pharmacotherapy, that the efficacy of medication is not dependent on age. For this particular trial, potentially unlike other disorders, there will be no targets for specific racial characteristics given that our previous trials recruit ethnic backgrounds that mirror the treatment seeking AUD population in Australia. Ethnic background will be recorded and presented in the main results manuscript.

3. Page 8 of 33, Patient recruitment and sample details: Is there a projected timeline for recruitment – a projected beginning and ending date? Will participants engage with the program on a rolling basis, as clients are enrolled, or will the program commence and conclude all at once with a full roster of clients?

AC: As stated in the Trial Status section on page 18, the expected recruitment timeline is ongoing until. The participants will be enrolled on a rolling basis which is now stated in the manuscript (2nd paragraph, page 5)

4. Page 11 of 33, line 52: I'm curious about the rationale for asking participants to defer concurrent psychotherapy for the first 6 weeks of the trial. Why 6 weeks? It's understandable to consider psychotherapy as an important modification to the treatment protocol, but I wonder how you will collect and analyze data around psychosocial and behavioral interventions in association with the prescription. I do not see it mentioned in the proposed analysis.

AC: Medical management is a form of light psychosocial intervention that is detailed in the protocol that all participants will receive. Given some evidence based therapy have evidence base for AUD this should not be commenced at the start of the trial. For the few that are referred to psychotherapy for duty of care reasons during the trial, this will be deferred until at least the 2nd half of the trial (given less chance of contamination) and these are then included in the analysis of covariates. This is stated in full on page 10, First paragraph. This is the protocol that we have used for many of our previous trial Morley et al 2018, 2024.

RC: 5. Page 16 of 33, lines 26-31: Is there a projected amount of drop out that you anticipate or a threshold that you are willing to accept?

AC: Page 13 in the Statistical Analysis paragraph of the Methods section states that "All available data from participants who took at least one dose of medication will be used to follow intention-to-treat principles." The drop out rate will be reported as per SPIRIT protocol guidelines.

6. The Discussion section is rather brief. It could be expanded to include alternative scenarios for the protocol, limitations, and research implications.

AC: We thank the reviewer for this suggestion. While discussion sections in protocol publications are usually brief, we have now included an extra paragraph (highlight in yellow on Page 14 in the discussion) discussing the strengths and research implications. There will be no alternative scenarios for the protocol listed until implementation informs otherwise. Similarly, limitations will be known upon and reported following completion of the trial.

Reviewer: 2
Dr. amir ghaderi, Kashan University of Medical Sciences
Comments to the Author:
The authors investigated Effects of "N-acetyl cysteine for the treatment of alcohol use disorder:
study protocol for a multi-site, double-blind randomized controlled trial (NAC-AUD study)". However, many points should be addressed in the revised version.

RC: What has been the basis for the classification of the groups?

AC: The introduction details thorough basis for choice of NAC as a medication of investigation. We thank the reviewer for this suggestion and we now have included comment regarding the choice of placebo group (Page 9, First Paragraph).
RC: What is the justification in your choice of N-acetyl cysteine dose?
AC: We thank the reviewer for this suggestion and we now have included justification regarding the choice of NAC dose (Page 9, First Paragraph).

RC: The keywords should be taken from the Medical Subject Headings.

AC: These have been reviewed.

RC: What is the criterion for choosing an age range of 18 to 70 years? Specify. Do not have this problem at an older age.

AC: International high quality prevalence in addition to Australian clinical treatment data definitely indicates that individuals have AUD at 70 years of age.

RC: Is there a precise documentation of side effects following international standards? Please provide this information.

AC: This is a protocol paper not the main effects paper. Thus, the side effects are not documented in this manuscript.

RC: Did not people study smoking and drug use? Do not use hypnotic drugs? Do you think these do not interfere with the study? How are they controlled?

AC: This is a protocol paper not the main effects paper. Thus, the side effects are not documented in this manuscript. As listed in the randomisation paragraph in the Methods section (Page 7) those with tobacco use will be stratified. Those with substance use disorder are excluded and those with psychotropic agents are excluded also (except from

antidepressants stable for 2 months). These are all detailed in the Inclusion/Exclusion criteria section on Page 8.

RC: No information about the physical activity records.

AC: This is a protocol paper not the main effects paper. Thus, physical activity is not documented in this manuscript. We list the measures we use in the trial (including International Physical Activity Questionnaire – Long (IPAQ-L) however we cannot provide the information given this is a protocol paper with no results.

RC. The table result is unclear. Please state the changes between the two groups in the variables examined, also β (95% CI).

AC: This is a protocol paper not the main effects paper. No results are presented in this manuscript.

RC. In the discussion, refer to the new article published in this field." Padoei, Fateme, et al. "The therapeutic effect of N-acetylcysteine as an add-on to methadone maintenance therapy medication in outpatients with substance use disorders: A randomized, double-blind, placebo-controlled clinical trial." Brain and Behavior 13.1 (2023): e2823".

AC: We have now referred to this article in the Introduction (see page 3, last paragraph).

10. Please describe the statistical analyses in more detail. For example, did you adjust the outcome variable measured in the follow-up measurements for the baseline value of the outcome (according to the equation $Yt = \beta 0 + \beta 1 * X + \beta 1 * Yt0$, where Yt = the outcome measured in the two follow-up measurements, X = treatment variable, β1 = overall treatment effect, and Yt0 = outcome variable measured in the baseline measurement)? Please indicate the parameters you have adjusted for in your linear regression analysis. It is well acceptable that an appropriate significance level α, such as 0.05, is pre-specified to guarantee the probability of incorrectly rejecting a single test of null hypothesis (H0) no larger than α. However, there are many situations where more than one or even a large number of hypotheses are simultaneously tested, which is referred to as multiple comparisons. Because you are testing many different hypothesis simultaneously ("multiple comparisons"), proper adjustment of statistical inference is required.

AC: This is a protocol paper not the main effects paper. No results are presented in this manuscript.

RC. Did the drug (NAC) have side effects in obese adults?

AC: This is a protocol paper not the main effects paper. No results are presented in this manuscript.

RC. Identify the primary and secondary outcomes.

AC: This is a protocol paper not the main effects paper. No results are presented in this manuscript.
